# Data Shepherding in Nanotechnology. The Initiation

**DOI:** 10.3390/nano11061520

**Published:** 2021-06-08

**Authors:** Irini Furxhi, Athanasios Arvanitis, Finbarr Murphy, Anna Costa, Magda Blosi

**Affiliations:** 1 Transgero Limited, Cullinagh, Newcastle West, V42V384 Limerick, Ireland; Finbarr.murphy@transgero.eu or; 2 Department of Accounting and Finance, Kemmy Business School, University of Limerick, V94T9PX Limerick, Ireland; 3 Department of Mechanical Engineering, Environmental Informatics Research Group, Aristotle University of Thessaloniki, Box 483, 54124 Thessaloniki, Greece; tharvanitis@meng.auth.gr; 4 Institute of Science and Technology for Ceramics, National Research Council of Italy (CNR-ISTEC), Via Granarolo 64, 48018 Faenza, Italy; anna.costa@istec.cnr.it (A.C.); magda.blosi@istec.cnr.it (M.B.)

**Keywords:** nanotechnology, data stewardship, data shepherd, FAIR, data management

## Abstract

In this paper we describe the pragmatic approach of initiating, designing and implementing the Data Management Plan (DMP) and the data FAIRification process in the multidisciplinary Horizon 2020 nanotechnology project, Anticipating Safety Issues at the Design Stage of NAno Product Development (ASINA). We briefly describe the general DMP requirements, emphasizing that the initial steps in the direction towards data FAIRification must be conceptualized and visualized in a systematic way. We demonstrate the use of a generic questionnaire to capture primary data and metadata description from our consortium (data creators/experimentalists and data analysts/modelers). We then display the interactive process with external FAIR data initiatives (data curators/quality assessors), regarding guidance for data and metadata capturing and future integration into repositories. After the preliminary data capturing and FAIRification template is formed, the inner-communication process begins between the partners, which leads to developing case-specific templates. This paper assists future data creators, data analysts, stewards and shepherds engaged in the multi-faceted data shepherding process, in any project, by providing a roadmap, demonstrated in the case of ASINA.

## 1. Introduction

The current digital innovation challenge is to link emerging technologies by bridging datasets across domains to consumer needs and societal demands [1]. In academia, much like in industry, data are rarely leveraged beyond their original intended purpose [2]. Technologies that produce data which meet the principles of Findability, Accessibility, Interoperability and Reusability (FAIR) are fundamental enablers for digital transformation [3]. These principles were coined in 2014 as a set of minimal guiding rules and practices for research data stewardship in the life sciences [4]. On the other hand, the FAIR principles do not provide specific guidance on data creators (experimentalists) on how to FAIRify their data, being heavily technical in nature [5]. The European Union (EU) has released guidelines on FAIR data management in Horizon 2020 (H2020) projects [6] and included the H2020’s Data Management Plan (DMP) as an intrinsic deliverable of any H2020 project that generates, assembles or processes research data. In addition, the EU provides Open Research, a scholarly publishing platform available to H2020 and Horizon Europe beneficiaries free of charge to promote reusability (https://ec.europa.eu/info/research-and-innovation/strategy/goals-research-and-innovation-policy/open-science/open-access_en, accessed on 1 May 2021).

A DMP includes diverse components regarding data, such as data and metadata summary, FAIR data, allocation of resources, data security, ethical aspects, and other issues. As an overall practice, data management is connected with the entire lifecycle of data implementation, including the primary steps of data creation, capture, variations and final storage [1]. DMPs facilitate the above aspects, as they play a major role in data FAIRification. As part of making research data FAIR, a DMP should consist of information on:

- the handling of research data during and after the end of the project;
- what data will be collected, processed and/or generated;
- which methodology and standards will be applied;
- whether data will be shared/made open access;
- how data will be curated and preserved (including after the end of the project).

The FAIR principles refer to a number of features that data should have to maximize their value, summarized under the umbrella terms of data being Findable, Accessible, Interoperable and Reusable. The four higher principles of FAIR have already been described in great detail in previous papers [3,4,7,8]. FAIR data principles have been developed to define good practices in data management. What constitutes “good” data management or stewardship under the FAIR principles is, however, still not clearly defined, and is generally left as a decision for the data owner [7,9]. This is why Papadiamantis et al. [5] tried to define the scientific FAIR principles to complement the highly technical FAIR principles and provide guidance to data creators on the steps required to FAIRify their data with extensive metadata capturing and the use of persistent identifiers.

Researchers who are unfamiliar with the concept of data management can find it difficult to operationalize the criteria and convey the requirements to others. Despite its significance, metadata capturing, for example, is not widely implemented in everyday academic scientific practice. This is due to the lack of data management training and the general understanding of data management as something that occurs at the end of a project, after data are thoroughly analyzed and published [5].

While certain fields generate data that are less problematic in terms of comprehension, format and sensitivity, other fields may face material and ethical concerns on data preservation. Nanosafety research is rapidly transforming into a highly competitive and data-intensive field [10,11]. In the field of nanoscience, data management is both a crucial opportunity and a challenge in terms of capturing and integrating knowledge from various fields (i.e., material scientists, toxicologists, chemists, regulators, etc.) [12]. Making data compliant with the FAIR principles is still at a trial phase regarding which criteria should be met first and how [13].

The previous work in the FAIR field addresses various aspects that lead to high-quality datasets, such as focused nano-curation workflows, evaluation of the quality and completeness of curated data, and database integration [5]. Jacob et al. [13] illustrated with experimental nano-data tables an approach (associated with a Design of Experiments) that can serve as a model for data management that enables data to be disseminated while meeting the FAIR criteria. Papadiamantis et al. [5] provides two case studies; the first addresses dose metrics and the prediction of nanoparticles (NPs) agglomeration to determine a delivered dose that correlates with toxicity. The second case study considers the need for agreed and harmonized terminology as a means to comprehend the role of NPs dissolution as a driver of toxicity. Some examples go beyond the FAIRification process and capture concepts such as:

- Data integration, which is one of the primary steps to generate high quality data for modeling purposes [14,15,16]. Data integration challenges emerge late at the time of analysis and reuse; the best point of action is at the point of data collection;
- Machine learning (ML) FAIRification supporting the end-to-end reproducibility of ML pipelines. Samuel et al. [17] investigated which factors beyond the availability of source code and datasets affect the reproducibility of ML experiments and proposed ways to incorporate FAIR data practices into ML workflows;
- Evaluation of FAIRness [18]. It is often useful to determine to what extent a resource (data or metadata) adheres to the FAIR principles. A number of different initiatives are currently focused on defining frameworks, approaches and criteria for assessing FAIRness. Thompson et al. [19] captures a variety of initiatives, as well as a number of available online evaluation tools.

While the above examples provide excellent resources for FAIR realization, they lack explanations for the initiation process of data collection among partners. The lifecycle of the data rising from multidisciplinary fields, from their generation to capturing and FAIRification, is supervised, facilitated and performed by the newly defined role of data shepherd [5]. The shepherd considers the needs of different users such as data creators (experimentalists who design and generate new data) and data analysts (the person who performs manipulation and analysis using computational tools) and combines the knowledge and insights in order to communicate requirements between the parties. In other words, a data shepherd is an extended version of a data steward, who only supervises the data management. In other words, the shepherd is strongly engaged and acts as a mediator of information flow between stakeholders by assembling experimental, computational and technical backgrounds. In addition to the communication aspect, the shepherd also leads the FAIRification process and is involved at a technical level (development of templates, annotation and curation), while spreading the awareness of the value of FAIR data.

In this paper, we detail a pragmatic approach to initiating the DMP and the data FAIRification process using the H2020 nanotechnology project, Anticipating Safety Issues at the Design Stage of NAno Product Development (ASINA). ASINA uses the production of two representative categories of nano-enabled products (NEPs), coatings in environmental nanotechnology (Category 1) and nano-encapsulating systems in cosmetics (Category 2), to formulate the design hypothesis and make design decisions by applying a data-driven approach and methodology. The methodology encompasses distinct phases to deliver Safe-by-Design (SbD) solutions. Designing new NPs or NEPs that compete in the market necessitates meeting a variety of performance criteria, such as improved functionality, cost-effectiveness, environmental sustainability and safety, which is a scientific challenge requiring the involvement and participation of people with different expertise. The ASINA SbD approach is based on a modular framework mapping NEPs’ life cycle with four modules: (1) synthesis, (2) processing and incorporation, (3) use phase, and (4) end of life/recycling.

ASINA offers an excellent opportunity to describe how complex data from multidisciplinary sources can be captured, communicated among partners, and FAIRified. This paper is one in a series of articles on the DMP and FAIR data in the ASINA project. It represents the rationale and importance of a defined workflow for curation purposes that facilitates the ideation and concretization of actions and communications among partners. This article describes how this process was applied to initiate data capturing and our reflections in establishing this workflow. The roadmap described here is applicable to any project that generates, computes or handles data.

## 2. Materials and Methods

**Figure 1:**
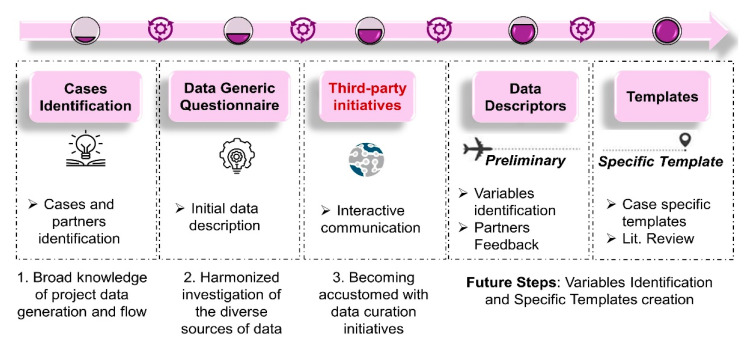
General workflow. Roadmap to final templates creation to be FAIRified for fit-on-purpose-specific cases.

Traditionally, the building blocks of data management are data templates. In ASINA we chose to create case-specific templates that cover the different aspects of the work to be performed and are able to capture all the necessary metadata to make the data fully understandable. These templates are then annotated and indexed to support human and machine data discovery and understanding. The first step, before any data capturing, is to establish a strong and clear communication with all project partners and acquire the knowledge and information to facilitate the data template design. Figure 1 demonstrates the roadmap for the template creation. A focused effort in each step in the workflow facilitates the identification of critical elements within the step.

Initially, project cases to be captured are comprehended and identified, usually from the Grant Agreement and presentations made among the consortia. The relevant data creators and analysts are also identified in this step. A broad knowledge and a holistic visual conceptualization of data to be generated and the flow within is required (see Section 3.1).

A questionnaire for an initial data description follows, which is circulated among the relevant partners previously identified (see Section 3.2). This step allows the data shepherd to gain an additional level of comprehension in the process.

Next, an interactive communication process is performed among the DMP responsible personnel and, external to the project, FAIR data initiatives. In our case, we initiated a Transnational Access application with the H2020 e-infrastructure project NanoCommons (see Section 3.3).

The next step includes the identification of data descriptors that define the variables of interest based on the partners’ initial feedback to the questionnaire. The preliminary template is created and circulated among the relevant partners to collect additional feedback regarding the dataset (see Section 3.4).

Finally, a detailed exploration of additional variables that “complete” the template is included and shared with the relevant partners (see Section 3.5).

Notice that the final two steps are data-oriented. At previous steps, the content may vary, but the workflow is the same across diverse subjects. It is at those two finals steps that the template design starts to differentiate among project goals and datasets. For this reason, those steps will be captured in detail in the series of papers that will follow.

## 3. Results

### 3.1. Cases Identification

During the ASINA lifespan, several data sources are foreseen to become available to different partners across a variety of expertise.

In more detail, experimental and computational procedures will be followed for a portfolio of candidate NPs, e.g., TiO_2_, nitrogen-doped TiO_2_ (TiO_2_-N), TiO_2_-SiO_2_, Ag, Ag-HEC (Hydroxyethyl cellulose) and synthetic or natural polymers as active carriers (encapsulation) such as Cr (Chromium-hydrolyzed wheat bran) and QV (Coenzyme Q10 and Vitamin E) incorporated into NEPs, regarding:

- data from the characterization for the identification of physicochemical (p-chem) characteristics, e.g., particle size and surface properties, chemical substance information;
- data from the assessment of the functionality, i.e., antibacterial, antimicrobial, photocatalytic or antioxidant capacity and of the cost-effectiveness (e.g., costing analysis and financial parameters);
- exposure assessment for workers, consumers and the environment, e.g., release, fate processes, exposure routes (life cycle assessment data);
- assessment of biokinetics such as bio-durability, uptake and dissolution rates, sedimentation, and impaction in the airways or airway bifurcations (internal fate estimation data coupled with modeling);
- human nanotoxicological data through in vitro assays (such as inhalation, intestinal, skin cell cultures), alternative in vivo toxicity (zebrafish), Adverse Outcome Pathways framework;
- eco-nanotoxicological data (*Enchytraeids*, *Collembola*, *C. elegans*, *Daphnia magna,* etc., species sensitivity distribution);
- field monitoring campaigns for exposure assessment in pilot plants, using online particle sizers and diffusion chargers providing time-series data that enable exposure modeling;
- Life-Cycle Inventory (LCI) of material, energy, environmental release inputs for each production unit and assessment of the resulting environmental impacts.

**Figure 2:**
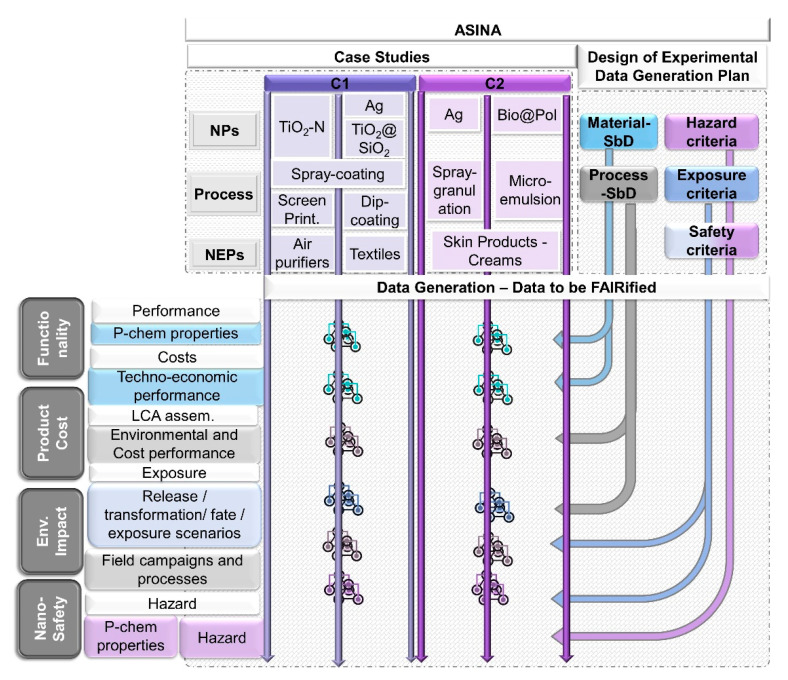
Mapping of case studies including the nanomaterials, processes and products in ASINA. C1 (Category 1) of anti-microbial coating in clean technology and C2 (Category 2) of nano-structured capsules delivering active phases in cosmetics. The design of experimental data generation represents a plan which will allow the objectives of the project to be met. Based on the design, new data across the life cycle production chain will be produced capturing a variety of dimensions around nano-production. This structure allows us to identify categories of future data to be generated and clustered into pillars. The mapping constitutes the first step towards the FAIRification workflow development.

An initial screening of the experimental/computational objectives suggests the creation of diverse datasets to capture different phases of the NPs/NEP life cycle. However, from Figure 2, the following information pillars are distinct: (1) *functionality*, which includes details of p-chem characterization coupled with life cycle costing performance indicators; (2) *nanosafety* (hazard analysis and exposure information across the life cycle); (3) *product cost* (techno-economic indicators and costs performance) derived from a life cycle assessment; (4) *environmental impact* derived from exposure assessment and scenarios and environmental toxicological studies. In each pillar, several partners with different stakeholder roles and expertise across different organizations and institutions are involved.

What is conceptually derived from Figure 2 has been addressed intrinsically in the ASINA design, where four corresponding pillars are identified for the final project objective (Safe-by-Design solutions). Integration of information into the four pillars represents one of the final steps of the ASINA project, but it is facilitated early on by the FAIRification process.

### 3.2. Initial Data Description—Questionnaire

In this second stage, the data shepherd creates a list with data creators/analysts in order to facilitate communication within the project and streamline data production and sharing, and in this way avoid confusion, frustration and delays among the other partners. A questionnaire is prepared and circulated among the relevant stakeholders to provide a first description of the data to be generated experimentally or simulated and the protocols and standards followed (Table 1, Response to Questions 2 and 3 of the DMP: what data will be collected, processed and/or generated and which methodology and standards will be applied). To facilitate information sharing and re-use, a private server has been set up on the ASINA project website (https://www.asina-project.eu/, accessed on 1 May 2021) for organizing information and initiatives of practice (Response to Question 1 of the DMP: Information on the handling of research data during the project). The questionnaire allows inner-communication among the partners involved in data generation, data management and curation, comprehension and data shepherding. Practically, it prototypes the generation of templates for data capturing. This step defines the beginning of the process of acquiring generic to specific input feedback from partners as well. To facilitate the initial description, we established a minimal information set to be included in the questionnaire (Table 1). The questionnaire is partially inspired by the PortForward project data management plan (version 1) (https://www.portforward-project.eu/wp-content/uploads/2018/09/PortForward-WP9-D9.5-Data-Management-Plan-V1-Final.pdf, accessed on 1 May 2021). Notice that the same approach can be used in diverse projects and its applicability has a broader context not limited to nanotechnology studies.

The questionnaire captures generic information, such as the following.

- Data identification: This part includes dataset description, which is a general description of forthcoming data (e.g., characterization, functionality analysis, cost effectiveness, life cycle analysis, fate in relevant matrices, toxicological data, exposure data, etc.). This will allow the generated data to be captured in different categories, harmonized and integrated. The source concerns whether the data are to be experimentally generated or simulated and the location of the analysis (e.g., partners lab or the software tool).
- Partner’s activities and responsibilities: This part includes personal information of the partners in charge of data collection/analysis and storage. In addition, related WPs and task(s) need to be captured in case communication or data integration is required.
- Expected input variables: For each analysis, some information will be needed in advance. For example, when in toxicological studies information on p-chem properties is required, data from particle characterization are necessary, and the template needs to be circulated among different/related partners. This part reveals which specific partners are related and how data are associated among WPs and/or tasks. It allows the capturing and exchange of data to be performed in a controlled manner while tracking additional contributions.
- Expected outcomes: This part is outcome-specific. Descriptions of the exact outcomes that are going to be produced are needed for the forthcoming integration of the pillars. Each WP/task, however, is expected to have several outcomes (for example, several toxicological assays will be performed for one NP). A description of each case should be recorded.
- Standards: Detailed description of the methods/protocols that will be followed to generate the data. This information will enable the data curation process and increase the FAIRness of data. The partners should describe which standards are applied for each case, and at this stage the data capturing structure is defined, informing partners and managers who might not be familiar with all the protocol applicability domains.

The questionnaire responses should be checked across the grant agreement and other materials shared by the partners (reports, presentations, communications, etc.) to confirm alignment with agreed upon project requirements.

Future steps require that the relevant partners inform the DMP’s responsible colleague for the sake of the confidentiality of the data (response to Question 4 of the DMP: whether data will be shared/made open access). A second questionnaire is then circulated to capture the information below (Table 2) and merged with the information in Table 1. Reasons as to why the data should have restricted access should be provided by each partner.

### 3.3. Collaboration with External FAIR Initiatives—NanoCommons

A great volume of nanosafety data generated from different participants in ASINA will benefit from becoming part of existing data curation initiatives. Based on the principle derived from coopetition, a Transnational Access (TA) (https://www.nanocommons.eu/ta-access/, accessed on 1 May 2021) application to NanoCommons (https://www.nanocommons.eu/, accessed on 1 May 2021) is the first step towards involvement with external initiatives. The TA ensures that datasets generated by ASINA are curated, harmonized, annotated and included in the NanoCommons Knowledge Base (https://ssl.biomax.de/nanocommons/cgi/login_bioxm_portal.cgi, accessed on 1 May 2021) (Response to Question 5 of the DMP: how data will be curated and preserved).

Annotation with recognized and established ontologies in collaboration with NanoCommons increases the FAIRness scores and the value of the data, and offers possibilities to future integration in available databases and data reuse under contexts different than those intended. This also allows the data to be combined with other datasets and exploited using existing and forthcoming computational tools in a scientifically accurate manner. This can lead to not only the production of more robust tools, but to the uncovering of hidden patterns via big data analysis. The eNanoMapper Ontology (currently maintained and developed under NanoCommons) includes various ontologies related to ASINA data, including the BioAssay Ontology (BAO), the Chemical Information Ontology (CHEMINF), the NanoParticle Ontology (NPO), the Relation Ontology (RO) and the Semanticscience Integrated Ontology (SIO) (https://www.nanocommons.eu/nanosafety-ontology-extension-characterisation-ecotoxicology-and-mesocosm-terms-added/, accessed on 1 May 2021). In collaboration with NanoCommons, those ontologies are assigned to the ASINA data. The shepherd collects the terminologies suggested by data creators and analysts, and searches in the abovementioned and other ontologies for corresponding concepts, as well as providing the semantic annotations and meta-data descriptions linked with ontological IDs. NanoCommons assures that the ontologies are in alignment with the knowledge base information within. Furthermore, this collaboration introduces the data into a number of activities within the NanoSafety Cluster (NSC) projects, currently active for the development of data dictionaries and ontologies, which are focussed on collection in specific fields of nanosafety research, such as exposure, hazard and p-chem characterization, for the grouping of these terms, and building hierarchies and relationships to translate these defined terminologies into ontologies (https://www.nanosafetycluster.eu/wp-content/uploads/Task%20Forces/NSC%20Ontology%20Task%20Force.pdf?_t=1601505310, accessed on 1 May 2021).

### 3.4. Data Descriptors—Preliminary Template

Different organizations incorporate common data formats in their respective workflows. An example of a data format is ISA-TAB-Nano, which is a file transfer protocol for querying among federated data repositories that are independently maintained by organizations with related, but not necessarily overlapping, objectives [12]. NanoCommons made suggestions related to data capturing, including the CEINT’s NanoInformatics Knowledge Commons (NIKC) Excel spreadsheet [12]. The CEINT-NIKC template was modified (simplified and streamlined via the H2020 NanoCommons infrastructure project) for use by the H2020 project NanoFASE, in order to capture complex mesocosm experimental data. Spreadsheets are a common format used by most professionals of all subfields of science, and due to their simplicity and familiarity, they are a reasonable choice for a data entry format.

**Figure 3:**
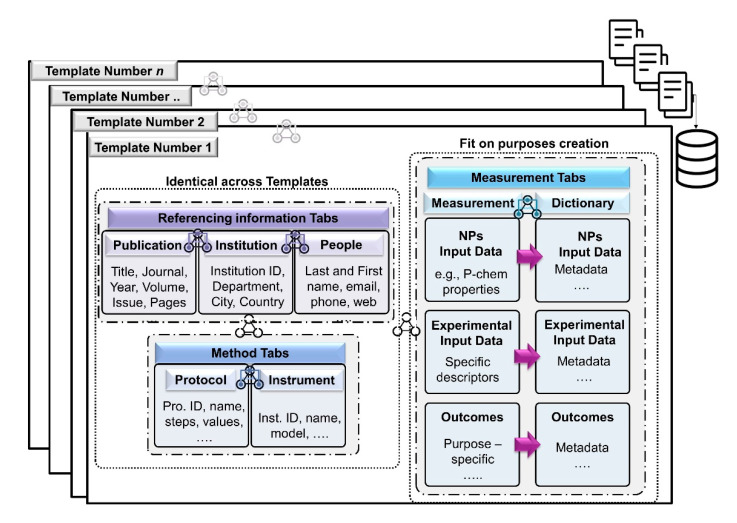
Dataset visualization. Tabs and variables included in each tab.

An Excel CEINT-NIKC template with seven spreadsheet tabs, including publication, institution, people, measurement, protocol, instrument and dictionary, is shown in Figure 3, and was selected to be used in ASINA to capture information regarding the data. These seven tabs are separated into three broad categories: reference information, measurement, and method. Information and examples regarding the structure of the template can be found here (https://wiki.nci.nih.gov/display/ICR/ISA-TAB-Nano+Curated+Examples, accessed on 1 May 2021).

The measurement tab is where experimental, raw data are deconstructed and organized for a specific ASINA case. Data are entered into this tab, but each data point is connected with metadata descriptions in the methods tabs (protocols and instruments) and annotated in the dictionary Table. Each new row entry into the spreadsheet is given a measurement ID. The data and metadata are linked together through the measurement ID in the referencing ID column. Every measurement ID is linked to a protocol and instrument ID when applicable, which allows the user to determine if the method used to achieve the results is relevant for their workflows or needs the addition of additional data points in computational analyses. The main benefit of the NIKC spreadsheet is that it can accommodate all types of nanosafety experiments, including simple computational workflows, although it is limited by the lack of a nanowide ontology for the annotation of terms, an issue being addressed currently within NanoCommons by expanding the eNanoMapper ontology [5].

### 3.5. Template’s Completion

The partners’ feedback to the questionnaire and the Excel spreadsheets comprise the primary steps in the data collection. A common file format, vocabulary and structure must be developed to facilitate the upcoming integration of the experimental information flow. The next step in the formulation of the case-specific templates is to identify particular variables that are required to be captured. The disadvantage of case-specific templates is that they are complicated and time-consuming to design and build. Finding a harmonized template for all data capturing among different fields, such as exposure assessment, toxicology, manufacturing processes, etc., without sacrificing data accuracy and clearness is not feasible.

The DMP partner must at this stage combine the external initiative spreadsheets with the information delivered by the project partners. The outcome is then shared with the data creators/analysts as the starting point of an interactive procedure towards the clarification and formalization of the data generation procedures. Information in the literature and data initiatives feedback supplements the process that is primarily driven by the partners’ feedback. The process is iterative until a consensus is reached between the data creators and other partners interested in the data, and the DMP partner is there to shepherd this communication. Future articles in this series will provide detailed explanations of the process, its implementation, and the variables captured in each case. They will also provide the final templates to be filled in. Further discussion of the detailed annotation and integration of knowledge is out of the scope of this paper but is already initiated at this stage. Annotation is performed in collaboration with NanoCommons and, when concluded, is the final step before data integration.

## 4. Discussion

This article explains and visualizes the process to initiate, design and implement data management and FAIRification processes in a multidisciplinary international project. To our knowledge, this type of task-flow log (case comprehension, composition and information circulation and interaction) has not been available yet in other projects implementing such processes. However, a similar concept to the questionnaire presented in this study has been established for the lifecycle of a metal halide perovskite experimental object, starting from the specification of an experimental set-up through the execution of each experiment object [20]. The authors mention that the lifecycle of an experiment starts in general as a structured, fill-in-the-blank form that provides a general pattern of information needed to specify an experiment. Each object is characterized by its particular type, material, action, observation, and outcome data. Treating entities in this fashion allowed the authors to track the experimental intent, reproduce the particular experimental execution, and clarify the relationship between the nominal experiment and the empirical observations. While the above article represents a concept based on a data model (software pipeline code) strictly applicable in experimental setups, we capture a general template for any type of data and incorporate the process of data shepherding as defined by [5], i.e., facilitating the communication and understanding of specific needs (experimental, analytical, computational) among different partners.

Professionals from various organizations with different skills, occupations and interests are brought together to explore data management and FAIR principles. However, in a multidisciplinary consortium, it is possible that some miscommunication and misunderstanding may occur. Thus, we investigate how the principles can be realized in practice, communicated, and also translated into final actions using a “data shepherd” who is able to understand, explain and guide the communication and capturing procedure. Our goal is to provide a roadmap to capture data among different specialties and partners, based on the non-technical scientific FAIR principles [5] that complement the original FAIR principles and explain the steps required by data creators without overcomplicating the concept. This case-specific paper focuses on the initial steps of creating an opening for guiding the multidisciplinary communication of FAIR data and the completion of a comprehensive DMP. DMPs are being promoted as open and discoverable resources to move towards a networked approach of data management. To date, this is supported by the DMPTool (https://dmptool.org/, accessed on 1 May 2021), DMPOnline (https://dmponline.dcc.ac.uk/public_plans, accessed on 1 May 2021), and by inviting authors of exceptional DMPs to publish them in Journal collections to achieve machine-actionable DMPs and to create a dynamic inventory of digital research-related outputs with shared benefits for diverse stakeholders [21]. While there are at the moment no protocols or standards to make nano-data FAIR, several efforts are underway [2]. If several projects reveal their actual processes towards FAIRification, such an act will accelerate the entire process and will narrow down the procedure into a more “Standardized way to make data FAIR in case where a universal template and ontology is missing”. Ultimately, data shepherding and data sharing are not ends in themselves, but means to more and better research.

External consultation from TA services facilitates and accelerates the curation process, especially in the first steps. Such initiatives should be nurtured until data FAIRification processes and culture are established among diverse stakeholders. When a critical mass of FAIRification implementations has been reached and training saturation has been achieved, further consultations will not be needed, and the consultative and educational TA services provided may transform into supervisory and assessing agencies.

Organizations or groups working to integrate or further improve a nanomaterial data curation workflow may benefit from adapting methods established in more mature fields (e.g., bioinformatics). In general, other fields utilize one of two approaches: (1) create unique file formats with standardized vocabularies, or (2) create collection formats at a generalized level to account for variance and uncertainty across a field. As a specific example of the first approach, the genomics community has developed a curation workflow that uses standardized file formats for both metadata and raw DNA sequence data for submissions into standard repositories [12]. As for the second example, according to GO FAIR (https://www.go-fair.org/, accessed on 1 May 2021), the steps involved are as follows: (1) retrieve non-FAIR data, (2) analyze the retrieved data, (3) define the semantic model, (4) make data linkable, (5) assign license, (6) define metadata for the dataset, and (7) deploy/publish the FAIR data resource [22]. While the aforementioned steps are clear, we here provide a transparent case/paradigm wherein the first steps of retrieving data are actualized and recorded.

The challenges and technical barriers included in the process of FAIRification are already stressed in the literature [2,3,5,9]. Regarding technical barriers, the successful deployment of FAIR across the entire life cycle assessment of the NEPs requires standardized information architectures. The most used nano-specific ontologies are the NanoParticle Ontology (NPO) and the eNanoMapper (eNM) ontology [5]. Nanofield should reach a robust consensus on the ontologies it uses to capture specific types of data [3]. Data annotation is a time-consuming and resource-intensive process posing a crucial challenge to overcome [3]. Choosing the correct concept, especially for researchers that have little to no experience with ontology mapping, is challenging, and performing ontology mapping for a complete data set is an extensive task [2]. To facilitate this process, some initiatives are created in order to find a common vocabulary. Specific to the SbD concept of ASINA, the EU NanoSafety Cluster Working Group E (WGE) on Innovation and Safer by Design (https://www.nanosafetycluster.eu/nsc-overview/nsc-structure/working-groups/wge/, accessed on 1 May 2021) leads an alignment effort on the selection of parameters (safety, performance factors, costs, sustainability considerations, etc.) and their contribution to decision-making. This initiative is shared by raw SbD projects, such as ASINA, SAbyNA (https://www.sabyna.eu/, accessed on 1 May 2021), SABYDOMA (https://www.sabydoma.eu/, accessed on 1 May 2021) and SbD4Nano (https://www.sbd4nano.eu/, accessed on 1 May 2021). Once related parameters are identified across diverse case studies, a common vocabulary is established naturally. Adapting the WGE outcomes to ASINA data management facilitates the communication of the data concepts included in the data pillars of ASINA cases, and strengthens the dissemination among other projects.

Regarding the challenges of FAIRification, research institutions are often not sufficiently equipped with the specific knowhow, and a number of researchers perceive this process as requiring additional paperwork that does not directly benefit their work. In our case, regarding feedback to the questionnaire, few of the partners failed to report specific information on data to be generated, and instead delivered descriptions of projects and literature reviews, or even how stakeholders could be involved. This was based on having a data shepherd who was able to explain to all partners the added value and the benefits derivable from a structured and harmonized capturing of the data produced. While review work is of high importance, this type of work cannot be considered as new data to be captured and FAIRified in the context of an industrial, highly technological project like ASINA. In this environment, a sufficient understanding of good data management, interdisciplinary skills, and effective communication are needed to gain an understanding of the objectives and problems of interested partners [5,23].

Another obstacle is the lack of interest in this task. In our case, very few partners failed to provide any feedback at all to the questionnaire. Additional resources requirements necessitate a change in the research culture, to move an organization from a protective data mentality to a mindset of data sharing and exploitation with all relevant stakeholders. Such a cultural change is best achieved via a combination of top-down commitment and investment from senior management with a bottom-up approach from scientists and managers. Data shepherds, charged with ensuring that good data governance is embedded and maintained within the organization, can catalyze this change [3], as they sit at the center of the data lifecycle and facilitate communication and understanding between all stakeholders [5]. In such a case, responsible data shepherding requires, in some cases, persisting and putting oneself in the difficult position of engaging top management while micromanaging technical collaboration at the project’s ground-base. Such management, however, is neither easy nor pleasant, yet it is necessary during the cultural transformation. A real-life ASINA case where multiple types of expertise are involved, with hands-on experience, contributes to realizing this aim.

Supplementary and significant to the above, training in FAIRification procedures, both technical and managerial, has to be conducted. The NanoCommons teams, in a joint initiative with the NSC, offer online educational webinars (https://infrastructure.nanocommons.eu/events/, accessed on 1 May 2021) on the concepts of computational tools, data annotation, FAIRification, ontologies, etc. However, it was not foreseen how the experience of the ASINA case could be consolidated into the training material. Disseminating similar experiences can trigger communication that will finally result in a dynamic, updated training continuity.

The upcoming papers in this series (DMP in ASINA) will reveal the journey among different stakeholders, how templates (measurement, metadata and dictionary tabs) are transformed through various case-specific requirements, and how the partners manage to fill them in with data (problems occurring; communication issues; literature gaps; variables to be captured; common vocabulary across the entire life cycle of nano production, etc.). This process is “alive” and delicate, and requires constant exploration and modification throughout the entire project’s duration. Our roadmap ends in templates that aid in creating a harmonized way of capturing data that decreases the time required for simplifying and optimizing a task that is highly time-consuming and requires the participation of several partners. A detailed description of the process followed up to the final templates is the first step towards the development of an automated method of template creation. For the moment, we foresee that each template generated will require excessive intercommunication procedures among partners. Every time a new variable has to be added to the template will require further communication. It is clear that an automated approach is required to facilitate this process by means of time and human resources, and will also facilitate data integration. While technology evolutions shift towards customer/producer entities, the digitalization of research processes is still not performed by infrastructures or IT systems, but by humans [23].

This is not just the case of ASINA; currently, the process to FAIRify data includes a number of manual steps that should be supported and automated [2]. “FAIR data” is in itself an abstract concept, and runs the risk of being perceived mainly in an IT-centric context [7]. However, for the moment, the idea is still in its infancy, and data shepherds are responsible for the majority of data-related issues. Data shepherds’ duties comprise ensuring data (and metadata quality) from a variety of sources interconnect with different “data stakeholders”, incentivize people to follow best practices, and enforce rules around data collection while helping to create and implement processes for data collection [3]. FAIRification, though, requires an enhanced version of a data steward, the data shepherd, who, also oversees dissemination in a wider group of parties, moderates conflicts, if needed, and guarantees the smooth flow of data upstream and downstream between roles, communicating effectively the usefulness and completeness of the (meta)data, even revising the metadata concept. Papadiamantis et al. [5] offers a detailed paper explaining the roles, such as data customers, creators, analysts, curators, managers and shepherds, and their responsibilities across the complete data lifecycle. They also define the scientific FAIR principles that complement the IT-centric FAIR principles, and explain the actions required from data creators in order to FAIRify their data. The latter research confirms the need for the new administrative role of data shepherd in data management. Although suggesting new, time-consuming managerial roles may seem obsolete, data shepherds are accountable for the majority of issues surrounding an extroverted management process for all data, and are, at least for the time being, necessary. Human involvement and caretaking are needed for establishing new practices and mentalities, while future research will focus on developing technologies to ensure the automation of the FAIRification process in future administrative roles.

## 5. Conclusions

This paper offers a roadmap for comprehending the initial actions required in the FAIRification process. It begins by capturing which knowledge is essential; then, it provides a questionnaire for capturing data descriptions from relevant data creators. It demonstrates the involvement of data curation initiatives, and offers a preliminary template to be transformed into case-specific final templates for curation. The paper gives direct examples from this exciting new area of “data management”, wherein all efforts will be weighed against the anticipated benefits of the digital revolution and evolutions in the nano field.

## Figures and Tables

**Table 1:** Form for collection of information on each dataset from the relevant partners.

Dataset Name	
Data Identification	
Dataset description	…..
Source	…..
Partner’s activities and responsibilities	
Partner owner of the data; copyright holder (if applicable)	…..
Partner in charge of data collection	…..
Partner in charge of data analysis	…..
Partner in charge of data storage	…..
Related WP(s) and task(s)	…..
Expected input variables	
Description of the information required (working packages (WPs) and/or tasks) in order to move forward.	…..
Expected outcomes	
Description of the specific endpoint measurement variables/outcomes.	…..
Standards	
Detailed description of the methods/protocols	…..

**Table 2:** Addressing data confidentiality among partners in the data management plan.

Dataset Name				
Data exploitation and sharing				
**Data exploitation (purpose/use of the data analysis)**	…			
**Data access policy/dissemination level:**	Public	…	Confidential *	…
**Data sharing, re-use, distribution, publication (How?)**	ASINA private server	…	ASINA private server	…
	CORDIS ^¤^	…		
	Publication (Embargo)	…		
	NanoCommons Knowledge Base †	…		
**Personal data protection: are they personal data? If so, have you gained (written) consent from data subjects to collect this information?**	…			
**Archiving and preservation (including storage and backup)**				
**Data storage (including backup): Where? For how long?**	…			

* Confidential: only for members of the Consortium and the Commission Services. ^¤^ CORDIS: The European Commission may publish the deliverable as well in the CORDIS platform. † NanoCommons: data will be curated and made available.
